# Evidence of the Amino Acids Tyrosine and Phenylalanine in the Interstellar Material of IC348 in Perseus

**DOI:** 10.3390/life15020181

**Published:** 2025-01-26

**Authors:** Susana Iglesias-Groth, Martina Marín Dobrincic, Antonio Pérez Garrido, Carlos Gutierrez

**Affiliations:** 1 Instituto de Astrofísica de Canarias, Via Lactea s/n, 38200 La Laguna, Tenerife, Spain; cgc@iac.es; 2 Physics Department, Universidad Politécnica de Cartagena, Campus Muralla del Mar, 30202 Cartagena, Murcia, Spain; fluctuacija@yahoo.com (M.M.D.); antonio.perez@upct.es (A.P.G.)

**Keywords:** astrobiology, astrochemistry, spectral line, identification, methods, laboratory, molecular, molecular data

## Abstract

We employed data from the Spitzer Space Telescope to investigate the presence of the aromatic amino acids tyrosine and phenylalanine in the interstellar gas of the young star cluster IC 348. Our analysis revealed emission lines in the observed spectrum that closely matched the strongest mid-infrared laboratory bands associated with tyrosine and phenylalanine in terms of wavelength and intensity. Through flux measurements, we estimated column densities along the line of sight toward the core of IC 348, ranging from 0.8–1.0 × 10^11^ cm^−2^. Additionally, these emission lines were evident in the combined spectra of more than 30 interstellar locations spanning various unrelated star-forming regions observed by Spitzer, indicating a widespread distribution of the molecules responsible for the emission throughout interstellar space. Prospective endeavors employing high spectral resolution mid-infrared searches for proteinogenic amino acids in protostars, protoplanetary disks, and the interstellar medium will play a pivotal role in elucidating the external origins of meteoritic amino acids and understanding the prebiotic conditions that laid the groundwork for life on early Earth.

## 1. Introduction

The presence of amino acids in meteorites has been recognized for decades, with numerous studies documenting their existence [1,2,3,4]. In carbonaceous chondrites, both proteinogenic and non-proteinogenic amino acids are notably abundant compared to other organic compounds [1,2,3,4]. Understanding their origin is pivotal for unraveling the emergence of life on Earth.

These meteoritic amino acids, along with other prebiotic molecules of biological significance, may have originated in the interstellar medium before the formation of the Solar System. Various mechanisms have been proposed, including the irradiation of icy mantles on dust grains, specific gas-phase reactions in dark clouds, and reactions involving protonated alcohols with formic acid (HCOOH) in the hot nuclei of molecular clouds [4,5,6,7,8]. These amino acids subsequently become part of the initial molecular inventory of protoplanetary disks in star-forming regions [9], potentially being incorporated into planetesimals that later form comets and asteroids, acting as precursors to deliver these molecules to the atmospheres of exoplanets during their early formation stages.

Remarkably, both proteinogenic and certain non-proteinogenic amino acids exhibit sufficient radiation stability to endure irradiation over billions of years due to radionuclide decay, when buried at specific depths in asteroids and comets (>20 m) and shielded from direct cosmic ray exposure [10,11,12,13,14,15]. Thus, the scenario involving amino acid formation in the interstellar medium, followed by their incorporation into planetesimals, asteroids, and comets, further supports the late delivery to Earth, as reinforced by these radiation chemistry experiments. However, there is growing experimental evidence suggesting that thermal and aqueous alteration processes may have influenced the distribution of amino acids—and their enantiomeric excess—found in carbonaceous chondrites [16]. Therefore, establishing the abundance of amino acids in the gas that are incorporated into proto-planetary disks of newly formed stars and setting the initial abundances of these molecules are critical for studying the impact of these processes.

Indeed, if amino acids are present in the interstellar gas of star-forming regions with abundances similar to those reported in meteorites [17], the absorptivity of their primary mid-IR transitions [18,19] may lead to their detection in the interstellar medium (ISM) using available observational facilities. A recent study [20] identified emission lines in the mid-IR spectrum of the interstellar gas of the star-forming region IC 348, consistent with the strongest vibrational transitions of tryptophan as measured in laboratory settings. This region, a young stellar cluster with an approximate age of 2 million years, lies at the eastern end of the Perseus Molecular Cloud, where previous studies have reported the presence of mid-IR bands of common molecules such as H_2_, OH, H_2_O, CO_2_, and NH_3_, along with several carbonaceous molecules that are potentially important for the production of more complex hydrocarbons, namely HCN, C_2_H_2_, C_4_H_2_, HC_3_N, HC_5_N, C_2_H_6_, C_6_H_2_, and C_6_H_6_. The derived abundances for HCN and C_2_H_2_, crucial molecules in the development of prebiotic building blocks, are on the order of 10^−7^ and 10^−9^ relative to H_2_, respectively. Additionally, more complex molecules such as PAHs and the fullerenes C_60_ and C_70_ are also present in this region [21]. IC 348 exhibits considerable richness and diversity in its molecular content, making it an ideal candidate for the exploration of complex organic molecules, including amino acids.

This study unveils findings from our research into the mid-infrared bands (10–33 μm range) of two aromatic amino acids, tyrosine (C_9_H_11_NO_3_) and phenylalanine (C_9_H_11_N_2_), within the gas of the star-forming region IC 348.

## 2. Materials and Methods

### 2.1. Tyrosine and Phenylalanine Laboratory Bands

**Figure 1:**
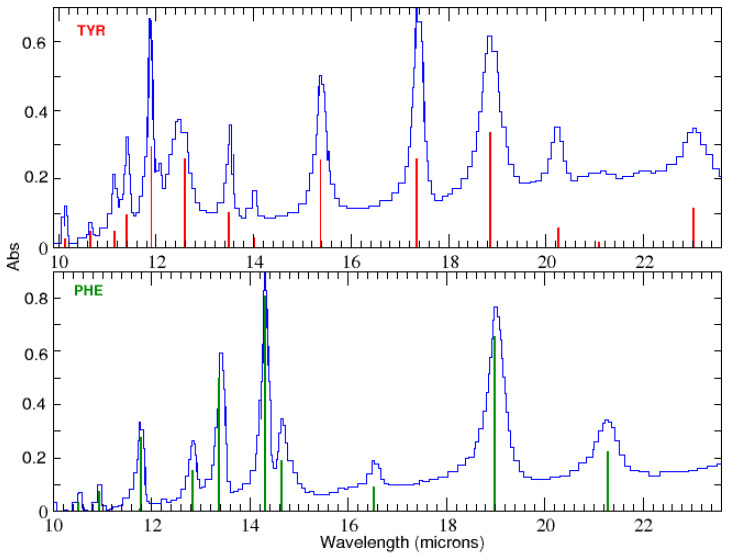
Mid-IR laboratory spectroscopy of tyrosine (**top panel**) and phenylalanine (**bottom panel**) from Iglesias-Groth and Cataldo [19]. Absorptivity is in relative units. The main lines are noted with vertical marks.

Systematic laboratory work (e.g., [18,22,23]) has led to the identification of many relatively intense amino acid bands in the mid-IR (Figure 1). Among the various proteinogenic amino acids, the aromatic amino acids tyrosine and phenylalanine display the largest number of bands reported between 10 and 36 μm. Absorbance is in relative units.

The main transitions are marked with vertical red lines with strength normalized to the peak absorbance of the strongest line at 18.87 μm for tyrosine and 27.32 μm for phenylalanine.

Matei et al. [23] obtained the vibrational spectrum in the transmission of amino acids mixed with polyethylene and pressed into disks, with a fast-scanning Fourier transform spectrometer. Their measurements, conducted at 25 °C, had a resolution of 1 cm^−1^. The technique used in the laboratory by Iglesias-Groth and Cataldo [18] to obtain mid-infrared spectra of tyrosine and phenylalanine at room temperature (see Figure 1) used a solid polyethylene substrate, beam splitter, and DTGS/polyethylene detector while the spectrometer was continuously pushed with dry CO_2_-free air.

Transmission IR spectroscopy was used on a solid sample obtained by compressing either CsI or PE powder with a solid matrix in a conventional press and die to granules. The wavelengths of these bands appear reliable and were measured consistently by various teams. Frequencies are consistent within 1 cm^−1^ and measurements of molar extinction coefficients and integrated molar absorptivity are available for all of them [19]. 

### 2.2. Observations

**Figure 2:**
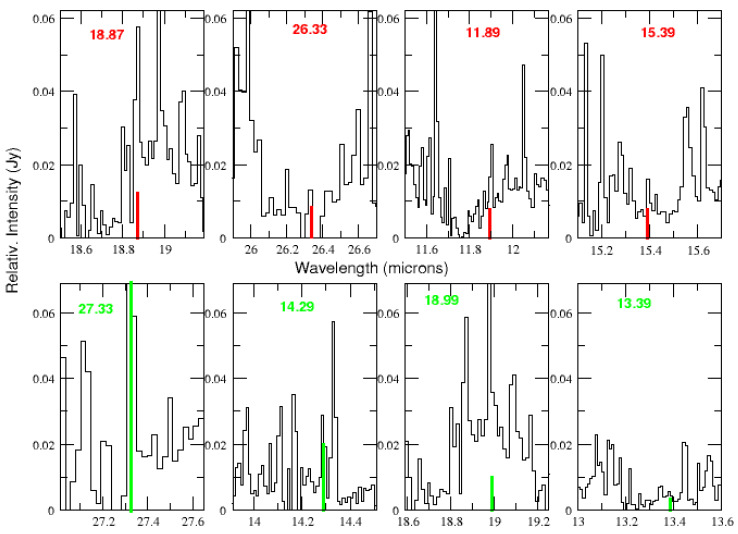
Selected regions of the mid-IR spectrum of the ISM in IC 348. Top panel: The four most intense laboratory bands of tyrosine are marked (red lines) with lengths proportional to the measured integrated absorptivity of the strongest line in the laboratory. Bottom panel: Same for phenylalanine (green lines). Both panels display the location of each amino acid line in a sequence of decreasing laboratory strength from left to right.

We have used moderately high spectral resolution (R ≈ 600) archive spectra obtained with the Infrared Spectrograph (IRS) onboard the Spitzer Space Telescope at various interstellar medium locations in the central region of IC 348. All the selected observations (Table 1) were located within 10 arcmin of the most luminous star of the cluster, HD 281159. The details of the observations and data reduction procedures are provided in Iglesias-Groth and Marin-Dobrincic [24] and their final combined ISM spectrum of IC 348 is used in this work. Selected regions of this spectrum are plotted in the various panels of Figure 2 where the wavelengths and relative strengths of the four strongest lines of tyrosine (top panel, red lines) and phenylalanine (bottom panel, green lines) are also marked.

Figure 2 shows the counterpart emission lines for each amino acid’s four strongest laboratory lines. The horizontal panels are displayed in a sequence of decreasing strength of the amino acid laboratory lines from left to right. The length of the vertical marks is normalized to the intensity of the strongest laboratory line of each amino acid. For every laboratory line, we find a tentative counterpart emission line in the observed spectrum that is consistent in wavelength and strength with the lines measured in the laboratory. The four strongest lines of tyrosine in the lab have tentative counterparts in the observed spectrum prompting the search for additional lines of this amino acid. In the case of phenylalanine, there is a remarkable decrease in the intensity of the lab lines, making the identification of any other weaker lines from this amino acid very difficult. One must keep in mind that we should expect differences between line widths observed in the ISM and those measured in the laboratory due to the differences in the environmental conditions—such as temperature, pressure, grain structures, and sizes—as these factors can cause line broadening.

Laboratory frequencies, wavelengths, and integrated molar absorptivity (*ψ*) for all the strongest (*ψ* > 1 km mol^−1^) mid-IR transitions of tyrosine and phenylalanine (obtained at a temperature of 20 °C) are listed in Table 2. Measurements of integrated molar absorptivity have uncertainties of the order 20%. In the following section, we present a systematic search in the interstellar spectrum of all the lines measured in the laboratory and examine possible contaminating lines from other molecules that may affect them. 

### 2.3. Data Processing and Spectral Lines

The uncertainties were estimated directly by measuring the RMS in the continuum of the final spectrum in areas less contaminated by lines. The uncertainties we claim, and subsequently the sensitivity of our final spectrum, are consistent with the expected sensitivity of the IRS according to the total integration time invested in the final spectrum. In addition to considering the physical effects of the medium on the observed spectra, other observational particularities such as low resolution, experimental equipment, and data manipulation processes must be accounted for to avoid confusing artifacts with possible emissions. Therefore, post-processing of observational data is equally, if not more, important than the observations themselves. The Spitzer Space Telescope data have undergone extensive iterative data correction, starting with bad pixel masks from subsequent observations.

Lebouteiller et al. 2015 [25] developed a method for optimal data treatment, including a differential technique that eliminates low-level rogue pixels without requiring dedicated background observations. This method has been applied to all observed data, expanding its usability. The Spitzer Heritage Archive (SHA) contains post-BCD data products for all Spitzer/IRS observations. However, the Atlas Cassis archive, used in this work, offers several advantages over SHA, including improved rogue pixel rejection. Bad pixels are initially cleaned using a rogue pixel mask calculated from all observations across campaigns. Remaining low-level rogue pixels are eliminated by one of two main methods: removing the observation at the other nod position (by-nod) or removing observations at the two nod positions in the other spectral order (by-order), inspecting background image quality, and selecting the best possible option based on signal-to-noise ratio through a posteriori comparison. The typical effect of these residuals is accounted for in our estimation of the RMS of the continuum as the representative RMS was obtained from the measurements performed in many spectral regions. If these residual effects were widely spread through the observed spectrum, their statistical effect would be accounted for in our determination of RMS. We checked the mask they provided to evaluate the effectiveness and indeed, for unknown reasons, some narrow artifacts (one-pixel spikes) of moderate strength comparable to those of the lines we are investigating remain in some spectra and they may affect the fluxes of some individual lines in the final averaged spectrum of IC 348, but we can quantify the magnitude of this problem.

We know the typical FWHM of the lines from molecular hydrogen, water, and other common molecules seen in the spectrum (these were extensively studied and published in MNRAS [24]). With this information, we performed a close inspection of the final IC 348 spectrum and identified all likely narrow spikes. Then, we determined its spectral density, which resulted in order 2 spikes of 1-pixel size per wavelength interval of 1 micron. Since the typical FWHM of a line is only 0.01–0.02 microns (from short to long wavelengths), these spikes are not so frequent as to cause a significant problem to our proposed amino acid line identification.

The probability of misidentification is only of one line out of ten proposed, being conservative at most, and two of these suggested lines of amino acids may be the result of a misidentification by a moderately strong enough uncorrected rogue pixel. Such misidentified lines are possibly the two deviating most from the fit in the excitation diagram. This should not be considered a major problem to invalidate the case that a significant number of lines may truly be explained by real spectral features, and they agree with the strongest mid-IR transitions measured in the laboratory for the two aromatic amino acids under discussion.

Line fluxes were derived from the combined IC 348 ISM spectrum by fitting a Gaussian profile and integrating the flux above a local continuum using the IRAF SPLOT routine. A straight line was used to fit the local continuum for a given emission line. Errors in those fluxes were estimated by propagating pixel error through the line integrals using the standard deviation of the residuals of the fit and are typically of the order of 20%. In Table 2, the uncertainties of each flux for individual entries are provided, along with indications if an emission feature coincides with lines from the molecules referenced by Iglesias-Groth and Marin-Dobrincic [24]. Upper limits were derived by measuring the flux of a Gaussian with a height three times the local RMS of the continuum, and with a FWHM equal to the instrumental resolution (approx. 0.02 μm). In those cases, where the RMS of the continuum was difficult to establish because of neighbor contaminant lines, the RMS was taken as that of the nearest region with a “clean continuum”, selected among the following spectral ranges: 10.10–10.15 μm, 11.75–11.80 μm, 14.35–14.5 μm, 14.60–14.7 μm, 15.45–15.50 μm, 16.80–16.90 μm, 20.40–20.70 μm, and 27.40–27.60 μm. These regions can be seen in Figure 2. The minimum flux level for a line detection in the combined IC 348 ISM spectrum resulted in an order of 1 × 10^−18^ Wm^−2^. Line detectability at such low fluxes opens the possibility of identifying weak transitions, enabling, in particular, a search for mid-IR transitions of the selected amino acids.

## 3. Results and Discussion

### 3.1. Tyrosine

**Figure 3:**
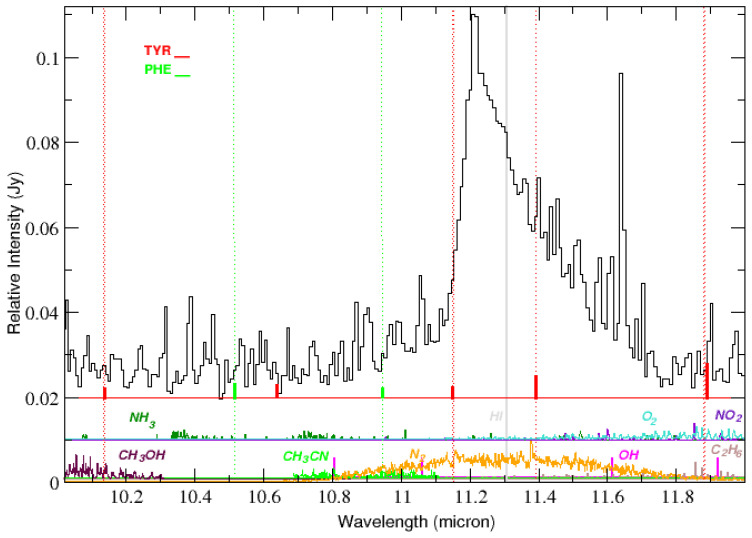
Emission lines in the IC 348 ISM spectrum (black) and laboratory bands of tyrosine (red dotted lines and vertical marks) and phenylalanine (green dotted lines and vertical marks) in the range 10.0–12.0 μm. The length of the vertical red and green solid marks corresponds to the relative strength of the tyrosine and phenylalanine lines as measured in the laboratory. The spectrum is dominated by the strong and broad PAH emission at 11.21 μm. At the two bottom panels, HITRAN synthetic spectra obtained at 240 K (the average temperature of the molecular gas in the region under study) are shown in different colors for various molecules. The synthetic spectra are scaled to obtain a good match of the strongest line in the computed spectrum of each molecule with its counterpart in the observed spectrum. The synthetic spectra of NH_3_, O_2_, NO_2_, CH_3_OH, OH, N_2_, OH, C_2_H_6_ are plotted in the same font color of its corresponding label and H I emission is marked at 11.309 μm (grey dotted line).

**Figure 4:**
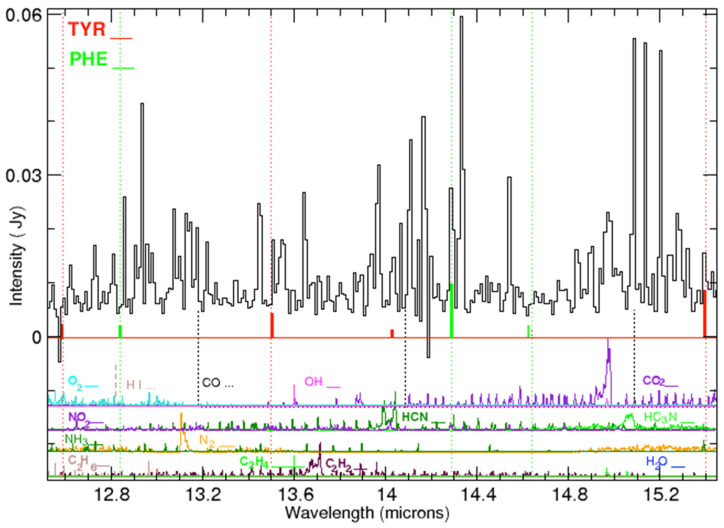
Emission lines in the IC 348 ISM spectrum (black solid line) and location of the laboratory bands of tyrosine (red dotted lines and marks) and phenylalanine (green dotted lines and marks) in the range 12.5–15.5 μm. At the bottom, HITRAN synthetic spectra obtained at 240 K are shown in different colors. Synthetic spectra: O_2_ (cyan solid line), OH (magenta solid line), CO_2_ (violet solid line), NO_2_ (violet solid line), HCN (thick dark green line) possible contaminant at 14.045 μm, HC3N (green solid line), NH_3_ (dark green line), N_2_ (orange solid line), C_2_H_2_ (brown solid line), H_2_O (blue line), C_2_H_4_ (green solid line), and CO (black vertical dotted line).

**Figure 5:**
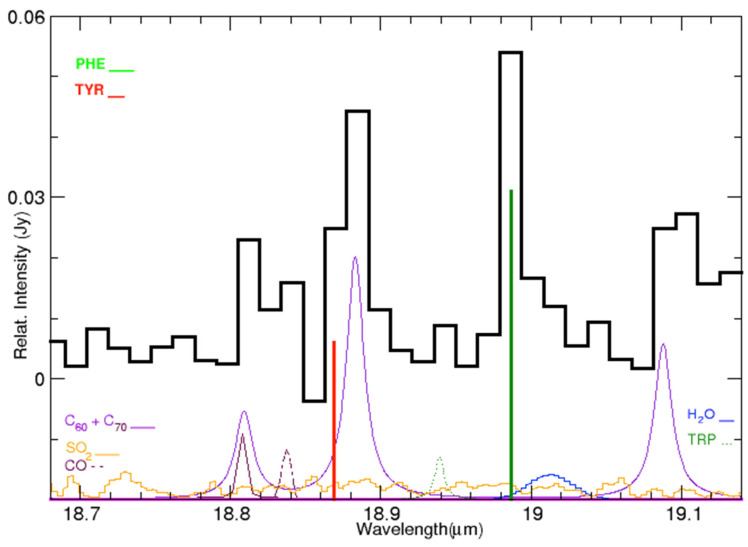
Emission lines in the IC 348 ISM spectrum (black solid line) and location of the laboratory bands of tyrosine (red) and phenylalanine (green) in the range 18.7–19.15 μm. At the bottom, HITRAN synthetic spectra obtained at 240 K; H_2_O (blue) with the line at 19.01 μm, OH (magenta), CO (black dashed), SO_2_ (orange). Lorentzians are used for the lines of fullerenes C_60_ and C_70_ (violet), and for tryptophane (TRP) at 18.94 μm (green dashed line).

**Figure 6:**
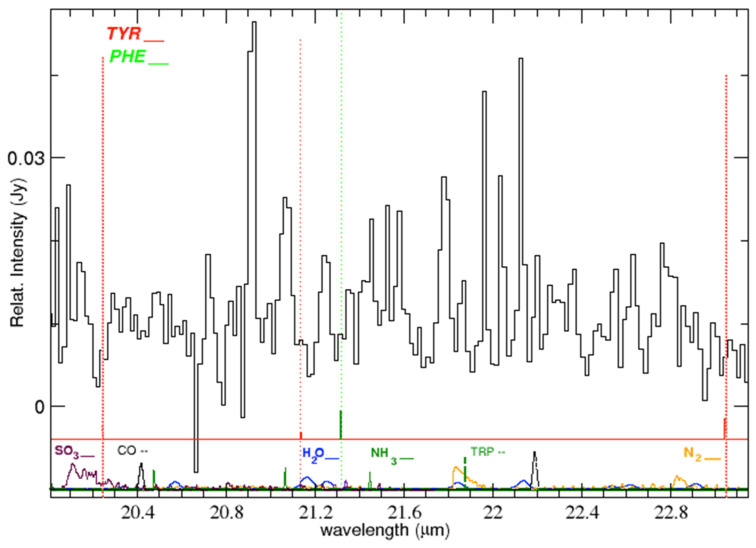
Emission lines in the IC 348 ISM spectrum (black solid) and location of the laboratory bands of tyrosine (red vertical marks and dotted lines) and phenylalanine (green vertical marks and dotted lines) in the range 20.0–23.1 μm. At the bottom, HITRAN synthetic spectra at 240 K for various molecular species. Each spectrum is plotted with the font color of its label. TRP designates tryptophan (green vertical mark).

**Figure 7:**
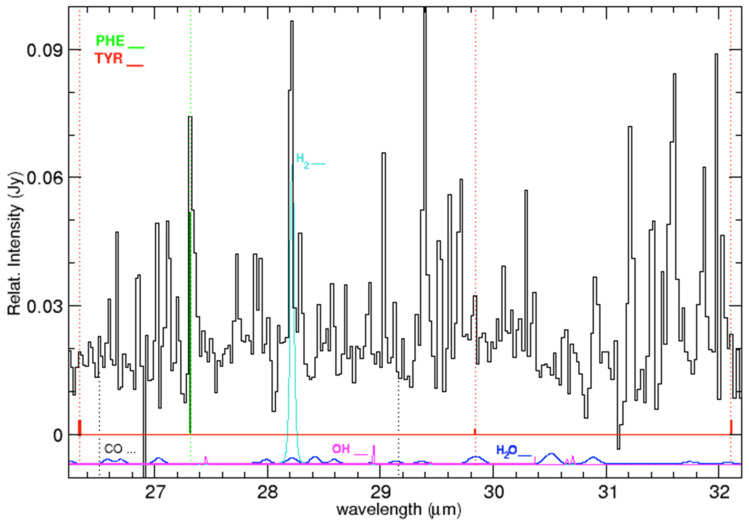
Emission lines in the IC 348 ISM spectrum (black solid line) and location of the laboratory bands of tyrosine (red vertical marks and dotted lines) and phenylalanine (green vertical marks and dotted lines) in the range 27.3–32.1 μm. At the bottom, HITRAN synthetic spectra at 240 K for various molecular species. Each molecular spectrum is plotted with the font color of its label.

In Table 2, for the range 10–33 μm, there are 17 tyrosine lines listed, together with their laboratory measurements of absorptivity. We found 16 emission features in the IC 348 ISM spectrum (see Figure 3, Figure 4, Figure 5, Figure 6 and Figure 7) that could be associated with these laboratory bands of tyrosine. The weakest laboratory band at 29.8 μm is clearly below the detection threshold in our spectrum. The observed wavelengths of the emission lines that are proposed as counterparts to the laboratory lines are also listed in Table 2. The same lines are marked in these figures together with their relative strengths and laboratory measurements of absorptivity.

### 3.2. Phenylalanine

In the spectrum of IC 348, we also identified several emission lines as potential counterparts for the phenylalanine laboratory lines listed in Table 2. The four most intense laboratory lines have counterparts at 27.32, 14.29, 18.98, and 13.39 μm, which coincide in wavelength and display relative intensities that correspond well to their relative integrated absorptivity. The weakest of these four laboratory lines at 13.39 μm is close to the detection threshold of our spectrum as can be seen in Figure 2. The next six less intense laboratory lines are too weak for reliable detection (they are below the detection threshold).

In Table 2, we list amino acid laboratory lines, wavelengths, and fluxes of emission lines proposed as tentative counterparts in the IC 348 ISM spectrum. Some of these lines could be contaminated by lines from other molecules as indicated in the column with remarks and by other unknown species. Future high-resolution mid-IR observations should clarify the extent of this contamination.

**Table 2:** Laboratory wavelengths and integrated molar absorptivity, *ψ*, for mid-IR transitions of tyrosine and phenylalanine in the range 10–33 μm and with *ψ* > 0.7 km mol^−1^. Wavelength and flux measurements for bands in the averaged IC 348 ISM spectrum. Notes: *M*: line frequency reported in the literature [23]; *: COO-rock/bend/wag vibrations, *x*: NH_3_ modes, *+*: CO*α* N−deformations, *s*: strong line, *w*: weak and *vw*: very weak line. Flux errors are of order 20%. Upper limits are at 2*σ* confidence.

Frequency Lab	Wavelength Lab	Wavelength Obs	Ψ	Flux	Notes
(cm^−1^)	(μm)	(μm)	(km mol^−1^)	(10^−18^ W m^−2^)	Matei Lab.
Tyrosine					
985	10.15	10.14	2.8	1.0	blend CH_3_OH and PH_3_
939	10.65	10.64		0.8	w blend
897	11.15	11.15	2.3	<0.9	blend PAH
877	11.40	11.40	4.6	1.9	ind
841	11.89	11.90	14.5	6.4	partially blended
794	12.59	12.59	12.7	1.1	indiv
740	13.51	13.51	4.9	2.5	vw
713	14.02	14.01	1.2		vw and blended with HCN
650	15.38	15.39	12.7	1.5	650 *^,*M*,*s*^
639	15.65		12.7	<1.5	639 *^,*M*,*vw*^ blended
576	17.36		12.7		575 *^,*M*,*s*^ blended with H_2_O and fullerenes
530	18.87	18.88	16.0	8.6	529 *^,*M*,*s*^ partially blended
494	20.24	20.24	2.7	1.4	493 *^,*M*^
474	21.10	21.13	0.63	0.3	473 ^*x*,*M*,*vw*^
434	23.04	23.05	5.5	3.0	433 ^*x*,*M*^
380	26.32	26.33	15.6	6.5	377 ^+,*M*,*s*^
335	29.80	29.81	1.3	0.5	335 ^+,*M*,*s*^
311	32.10	32.1	7.1	2.3	310 ^+,*M*,*s*^
Phenylalanine					
950	10.53	10.53	1.7	0.8	blend, NH_3_, C_2_H_4_
914	10.94	10.93	1.7	0.8	blend
860	11.63	11.63	7.2		blend
779	12.84	12.85	3.6	<1.6	blend
746	13.40	13.39	9.2	4.5	
700	14.29	14.29	19.8	10.0	
700	14.29	14.29	19.8	10.0	
683	14.64	14.65	2.3	0.6	blend
605	16.51	16.51	2.1	1.0	605 ^+,*M*,*w*^ blend with CH_3_
526	19.01	18.99	15.0	6.3	525 ^+,*M*,*s*^
469	21.32	21.32	4.9	1.9	469 ^*x*,*M*^
366	27.32	27.32	54.0	13	365 ^+,*M*,*s*^

## 4. Discussion

### 4.1. Excitation Diagrams and Equilibrium Temperatures

**Figure 8:**
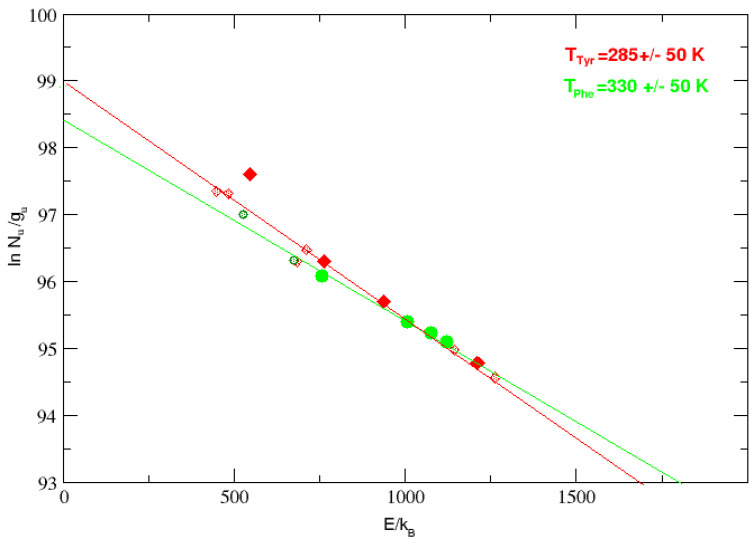
Vibrational excitation diagram for the lines tentatively assigned to tyrosine (strong lines: red solid diamonds, medium: shaded red diamonds) and phenylalanine (strong lines: solid green circles, medium lines: shaded green circles) in the spectrum of the ISM in IC 348. Weaker or blended lines are not represented. Natural logarithm of N_*u*_/g_*u*_ versus energy E_*u*_/k_*B*_ (cm^−1^) of the excited vibrational states for the observed amino acid bands with available fluxes and integrated molar absorptivity.

The vibration excitation diagrams for tyrosine and phenylalanine were obtained using the molar absorptivity of the strongest transitions free from obvious blends, in order to obtain reliable fluxes (listed in Table 2). The number of molecules in the upper vibrational state N_*u*_, for each transition, was obtained from the flux of each transition assuming optically thin emission. The total power emitted in a band (see, e.g., [26]) is P = N_*u*_ A_*ul*_ h*ν*_*ul*_/(4*π*D^2^), where D is the distance to IC 348 (315 pc), *ν*_*ul*_ the frequency of the amino acid transition, and h the Planck constant. The Einstein A_*ul*_ coefficients were obtained using laboratory measurements of integrated molar absorptivity. Since molar absorptivity coefficients are rather sensitive to temperature, vacuum conditions, and a vibrational degeneracy, g_*u*_ = 1 was adopted for all the energy levels; given the lack of information in the literature, we could only assume thermal equilibrium. However, the results plotted in Figure 8 (red diamonds for tyrosine and green stars for phenylalanine) show encouragingly good correlation coefficients about r = 0.9–0.95.

The inverse of the slopes of the fits in Figure 8 indicates equilibrium temperatures of 285 and 330 ± 50 K for tyrosine and phenylalanine, respectively, which are comparable to the excitation temperature found for H_2_ in the same region [24] and very close to the temperature adopted in the laboratory (290 K) for the measurement of the integrated molar absorptivity. The diagram in Figure 8 suggests that the tyrosine and phenylalanine molecules coexist in warm gas at the core of the stellar cluster. For comparison, in interstellar locations at the core of IC 348, similar procedures obtained equilibrium temperatures of the order 200–300 K for fullerenes C_60_ and C_70_ [21] and 290 ± 50 K for the aromatic amino acid tryptophan [20].

### 4.2. Column Densities

Under the assumption that tyrosine and phenylalanine are the main contributors to the reported emission lines, we can estimate column densities from the measured fluxes in procedures already used to derive abundances for other molecular species in the ISM of star-forming regions (e.g., [21,27]). The total IR intensity (Wm^−2^sr^−1^) emitted by amino acids can be estimated as I_*tot*_ = N_*ν*_(amino acid) × *σ*_*UV*_ × G_0_ × 1.2 × 10^−7^ where G_0_ is the radiation field in the locations of the observations and *σ*_*UV*_ is the cross section for absorption in the UV of the relevant molecular specie. Because of the presence of luminous stars in the core of IC 348, we will adopt G_0_ = 45 [20,21], where the value of G_0_ = 1 corresponds to 1.2 × 10^−7^ W m^−2^ sr^−1^. We measured fluxes only for the stronger lines in the 10–30 μm region. To estimate the total IR intensity, we adopted the fluxes predicted by thermal equilibrium computations for all the remaining laboratory IR lines of both amino acids. The total IR flux is considerably higher than the lump sum of the observed line fluxes, three times higher for tyrosine, and four times higher for phenylalanine. The estimated total emitted IR fluxes, considering these corrections, are 11 × 10^−17^ W m^−2^ and 9 × 10^−17^ W m^−2^. Line fluxes were converted into intensity (I_*tot*_) by dividing by the subtended area in the sky of the corresponding slit for each module of the IRS spectrograph. The amino acid UV absorption cross-section, *σ*_*UV*_, can be computed from laboratory measurements of the molar absorption coefficients *ϵ*(*λ*) using the relation *σ*(*λ*) = 1000*ϵ*(*λ*)/(N_*A*_log(e)) where N_*A*_ is the Avogadro number. The molar absorption coefficients *ϵ* (mol^−1^cm^−1^) for aromatic amino acids can be found in Wetlaufer [28] and Fasman [29]. The stellar radiation field at the core of the IC 348 cluster is dominated by the UV emission of the B5 and A2 most luminous stars in the cluster, with peak emission at 200 nm. Many cooler stars are present in IC 348 [30] but their radiation is dominantly emitted at wavelengths larger than 300 nm where tyrosine and phenylalanine have negligible absorbance, so, the most relevant wavelength range for determining the effective UV absorption cross-sections is from 190 to 230 nm, the resulting *ϵ*_*eff*_ are 5640 and 3530 mol^−1^ cm^−1^, and the corresponding mean UV absorption cross-sections are 2 × 10^−17^ cm^2^ and 1 × 10^−17^ cm^2^, for tyrosine and phenylalanine, respectively. The resulting column density for tyrosine is then N(tyr) = 8 × 10^10^ cm^−2^ and for phenylalanine N(phe) = 10 × 10^10^ cm^−2^ with the uncertainty of 50% in each case, mainly associated with our limited knowledge of the G_0_ parameter in the emitting region. We recall here that Iglesias-Groth [20] estimated a column density of 66 × 10^10^ cm^−2^ for the other aromatic amino acid tryptophan.

### 4.3. Comparison with Meteorites

Tyrosine and phenylalanine are systematically detected in carbonaceous chondrites. For instance, in CM2 types, their abundance levels are of the order 0.9–0.8 ppm. For comparison, glycine, the most abundant amino acid in any type of carbonaceous chondrites, is found with concentrations of the order 5–6 ppm (e.g., [17]). In IC 348, the fraction of gas phase carbon locked in tyrosine or phenylalanine can be estimated from their column densities via f_*c*_(AA) = N(AA) × n_*AA*_/N(H) × [C], where [C] = 1.6 × 10^−4^ is the carbon to hydrogen ratio in the region [31], n_*AA*_ is the number of carbon atoms in the amino acid molecule, and N(H) is the hydrogen column density, N; N(H) = 4.8 × 10^22^ cm^−2^ for the central part of the cluster IC 348 [32]. The result for tyrosine is f_*c*_(tyr) = 0.09 × 10^−6^ and for phenylalanine f_*c*_(phe) = 0.1 × 10^−6^. These fractions are very dependent on the derived column densities, which are very sensitive to the adopted G_0_. Nevertheless, these results are not far from the reported 0.9–0.8 ppm for these amino acids in several types of carbonaceous chondrites [3,33]. If the meteoritic abundance patterns hold for the gas in IC 348, then it shall be possible to detect the lines of other amino acids with higher meteoritic abundances as is the case of glycine.

**Figure 9:**
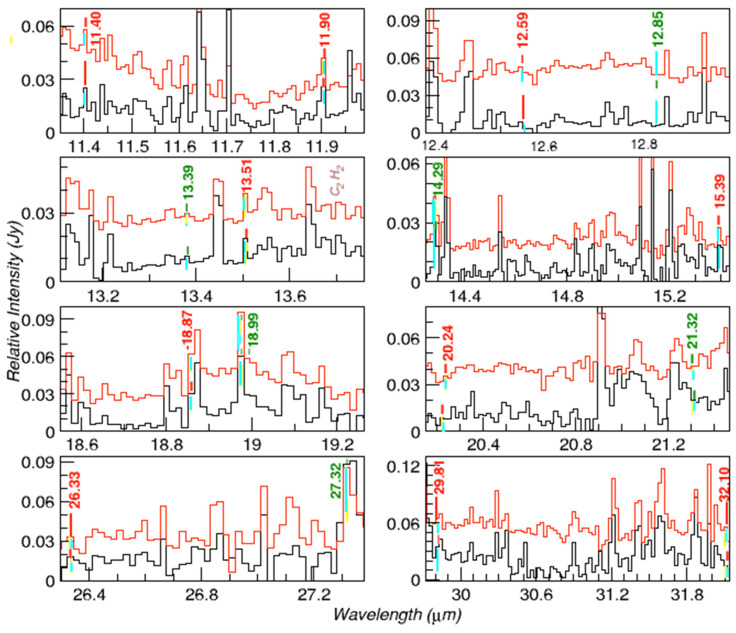
Comparison of the “combined IC 348 ISM spectrum” (solid red line) and the “combined star-forming region ISM” spectrum (solid black line) resulting from averaging spectra from various star-forming regions. The spectra are shifted on the vertical axis for convenience of display, and only band fluxes relative to the local continuum can be deduced from these plots. The wavelengths of tyrosine and phenylalanine bands are indicated with numbers in red and green, respectively. Emission features assigned to these bands are marked (blue/yellow filled). The brown label indicates the C_2_H_2_ line at 13.7 μm.

As we can see in Figure 9, the ISM IC 348 emission spectrum of the most relevant lines, considering their integrated absorptivity, proposed here as due to tyrosine and phenylalanine, are also present in the combined ISM spectrum generated from the combination of more than 30 spectra obtained in diverse unrelated star-forming regions. This reference spectrum is described in detail by Iglesias-Groth and Marin-Dobrincic [24]. Reliable column densities cannot be inferred from this combined spectrum because it is averaged from sources distributed in a large range of distances, and the average fluxes would not be meaningful. However, this reference spectrum displays the same bands we have assigned to amino acids with relative intensities like those found in IC 348. This could indicate that tyrosine and phenylalanine but also other amino acids detected in carbonaceous meteorites could be widely distributed across the Galaxy.

## 5. Conclusions

We have undertaken a search for mid-infrared transitions ranging from 10 to 33 µm of the aromatic amino acids tyrosine and phenylalanine. Utilizing Spitzer spectroscopic data, we have compiled a joint spectrum from various locations in interstellar medium (ISM) locations within the core of the IC 348 star-forming cluster. We pinpointed emission lines corresponding to the strongest laboratory bands for all identified bands, while also conducting a comprehensive study to identify possible contaminants. In the spectrum of IC 348, we identified bands of several molecules including H_2_, H_2_O, CO_2_, OH, C_2_H_2_, C_2_H_4_, C_6_H_6_, PAHs, and fullerenes. However, there remains the possibility of contamination affecting the emission features assigned to tyrosine and phenylalanine by other less common molecular species. Future spectroscopy with the James Webb Space Telescope (JWST) at higher resolution will validate whether tyrosine and phenylalanine are indeed responsible for the observed emission lines. Based on tentative band assignments to tyrosine and phenylalanine, we have constructed a preliminary vibrational excitation diagram, from which an equilibrium temperature in the range of 285–330 K is inferred (±50 K). Notably, the derived excitation temperature closely aligns with that determined for the more abundant molecular hydrogen within the same gas [24]. Additionally, by employing available UV absorption cross-sections and estimated total infrared fluxes, we have estimated tyrosine end phenylalanine column density in the ISM of IC 348, as well as the fraction of gas-phase-locked carbon in tryptophan. The fraction of gas-phase-locked carbon in IC 348 is lower than the fraction of amino acids relative to carbon typically observed in carbonaceous chondrites but consistent with the detection of tyrosine and phenylalanine in meteorites. If amino acids are indeed present in the ISM of star-forming regions, they could constitute a portion of the inventory of organic molecules in the protoplanetary discs. Thus, investigations in protostars and protoplanetary discs in Perseus and other molecular cloud complexes hold significance, as they may offer valuable insights into the delivery of complex organics by meteoritic and cometary material to planets in the early stages of formation. Ultimately, such studies could shed light on processes pertinent to the origin of life on Earth.

## Figures and Tables

**Table 1:** AOR of interstellar pointing, distance to HD281159 (LRLL 1), program ID (40247: N. Calvet and 50560: D. Watson).

AOR	Dist. to HD281159	Program	Object
*--*	arcsec	*--*	*--*
HR 22848512	204.14	40247	IC348-6 background
HR 22849536	322.86	40247	IC348-31 background
HR 22849024	340.21	40247	IC348-21 background
HR 22850048	391.6	40247	IC348-37 background
HR 22850560	571.94	40247	IC345-55 background
HR 22851072	566.82	40247	IC348-67 background
HR 22851584	592.16	40247	IC348-68 background
HR 27542016 (4)	81.37	50560	IC348-561263 + 32193111
HR 27542016 (1)	119.66	50560	IC348-561263 + 32193111
HR 27542016 (2)	119.66	50560	IC348-561263 + 32193111
HR 27542016 (3)	160.05	50560	IC348-561263 + 32193111

## Data Availability

The data underlying this article were derived from sources in the public domain and are available in CASSIS, the Combined Atlas of Sources with Spitzer IRS Spectra, at https://cassis.sirtf.com/. The final combined spectra can be provided under request to the author.
